# Oncogenic protein SALL4 and ZNF217 as prognostic indicators in solid cancers: a meta-analysis of individual studies

**DOI:** 10.18632/oncotarget.8237

**Published:** 2016-03-21

**Authors:** Ji Cheng, Jinbo Gao, Xiaoming Shuai, Kaixiong Tao

**Affiliations:** ^1^ Department of Gastrointestinal Surgery, Union Hospital, Tongji Medical College, Huazhong University of Science and Technology, Wuhan, China

**Keywords:** SALL4, ZNF217, prognosis, solid malignancies, meta-analysis

## Abstract

**Background:**

SALL4 and ZNF217 have been widely acknowledged as pivotal effectors stimulating embryonic immortalization as well as oncogenicity. Nevertheless, their prognostic worthiness towards solid tumors remains obscure. Hence we performed this comprehensive meta-analysis aiming to unveil the survival significance of both aberrantly expressed proteins.

**Results:**

Overall we included 22 eligible entries comprising of 3093 participants. Over-expression of SALL4 and ZNF217 were negatively correlated with clinical prognosis of 3-year, 5-year, 10-year and disease-free survival in solid malignancies, irrespective of cancer types, source regions, mean-age and sex predominance. Results of sensitivity analysis additionally verified the stability of the pooled outcomes. No publication bias was observed on the basis of Egger's test and Begg's test.

**Methods:**

Studies were eventually included via database searching and rigorous eligibility appraisal. Data extraction and methodological assessment were implemented under a standard manner. Review Manager 5.3 and STATA 12.0 were utilized as statistical platforms following the recommendations by Cochrane Collaboration protocols.

**Conclusions:**

Aberrant amplification of SALL4 and ZNF217 serve as unfavorable predictors of survival expectancy among cancer sufferers, revealing great potential as targeted spots in future therapeutics.

## INTRODUCTION

Over the past decade, the global healthcare system has been heavily burdened by soaring amount of solid malignancies [1]. Including surgical intervention and chemotherapy administration, resistance to current modalities primarily accounts for the growing mortality among cancer patients. Currently, targeted strategy has been regarded as a revolutionary breakthrough for cancer pharmacotherapies and refractory patients. However, partially due to inadequate prognostic evidences, targeted proteins involving in oncogenesis are not well characterized [2]. Therefore, discovery of novel spots for solid malignancies remains essential.

SALL4, expressively silenced in mature entities, is constitutively enriched in embryonic tissue and serves to maintain self-renewal capability [3, 4]. Aberrantly reemerging both *in vivo* and *in vitro*, SALL4 was first described as an oncoprotein in leukemia carcinogenesis [5]. Similarly, the oncogenic role of SALL4 has been subsequently confirmed among multiple types of solid malignancies [6]. Nevertheless, its predictive value in prognostication is limitedly reported, which significantly restrains its pharmaceutical prospects.

As a novel zinc finger transcription factor, ZNF217 was initially identified as a tumorigenic stimulator in breast carcinoma and was frequently amplified in diverse malignancies [7]. Accumulating evidences have linked its overexpression to increased chemoresistance and enhanced metastatic capability [8]. However, there is currently no direct evidence concerning the prognostic efficacy of ZNF217 in solid cancers, which has obstructed its clinical applications.

Therefore, as representatives of zinc-finger transcriptional factors with independent performances, we performed this comprehensive meta-analysis aiming to clarify the prognostic roles of SALL4 and ZNF217 in solid malignancies and provide promising targeted spots.

## RESULTS

### Demographic characteristics

**Figure 1:**
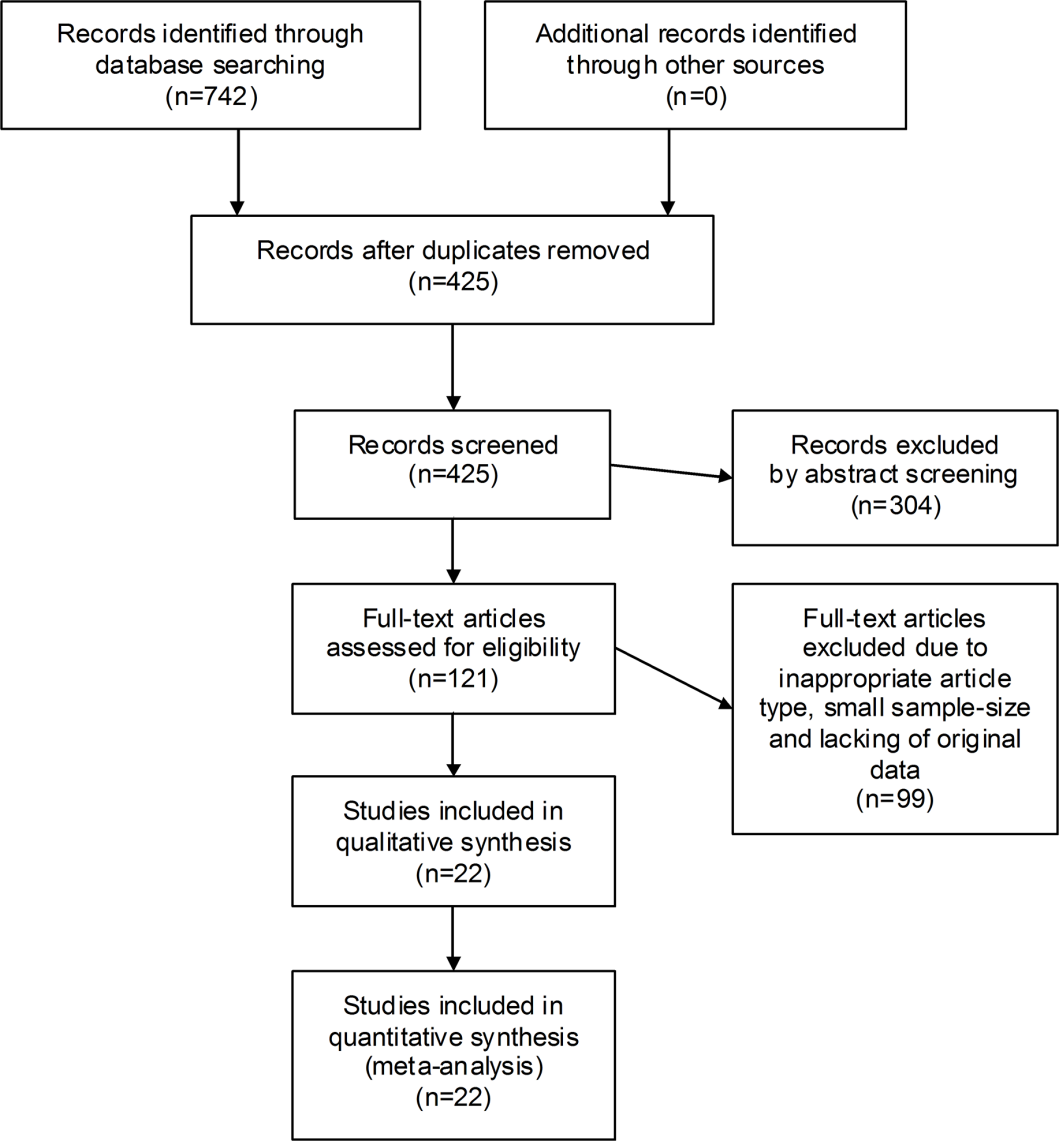
The flow chart of the selection process in our meta-analysis

Deriving from 742 preliminarily retrieved entries, 22 observational cohorts were eventually selected (13 for SALL4 and 9 for ZNF217), with a total sample-size of 3093 participants (2111 for SALL4 and 982 for ZNF217). The selection flow chart was depicted in Figure 1.

Among the studies featuring SALL4 expression, the chief source region and cancer type were Japan (*n* = 5) and liver cancer (*n* = 9) respectively. The wide spectrum of sample-size ranged from 38 to 337, with a median value of 144. Baseline parameters were statistically comparable between both contrastive groups. Additional details were demonstrated in Table 1.

**Table 1:** Demographic information for included studies with SALL4 expression

Reference	Country	Cancer type	No.	Mean age (y)	Male/Female	TNM stage	Median follow-up (m)	SALL4 (−/+)	Subcellular localization	NOS score
Deng et al 2015 [9]	China	Liver cancer	175	55.0 ± 13.6	93/82	NA	NA	73/102	Nucleus	7
Han et al 2014 [10]	China	Liver cancer	38	52.7 ± 12.5	35/3	I–IV	NA	20/18	Nucleus	6
He et al 2012 [11]	China	Ovarian cancer	90	61.8	All female	I–IV	88 (2–160)	62/28	Nucleus	7
Li et al 2015 [12]	USA	Endometrial cancer	113	57.3 ± 10.6	All female	I–IV	50 (0–143)	59/54	Nucleus	8
Liu et al 2014 [13]	USA	Liver cancer	236	62.0	165/71	NA	NA	233/3	Nucleus	7
Oikawa et al 2013 [14]	Japan	Liver cancer	139	57.0	102/37	I–IV	23	29/110	Nucleus	8
Osada et al 2014 [15]	Japan	Gastric cancer	92	63.4	46/46	I–IV	NA	68/24	Nucleus	7
Park et al 2015 [16]	Korea	Liver cancer	190	58.1 ± 11.8	151/39	I–IV	52 (0–133)	151/39	Nucleus	8
Shibahara et al 2014 [17]	Japan	Liver cancer	337	64.6	263/74	NA	0–48	290/47	Nucleus	7
Tanaka et al 2015 [18]	Japan	Liver cancer	90	NA	71/19	I–IV	NA	82/8	Nucleus	7
Yong et al 2013 [19]	Singapore	Liver cancer-SG	79	56.2	64/15	I–IV	98 (0–255)	34/45	Nucleus	7
		Liver cancer-HK	228	55.0	179/49	I–IV	75 (0–134)	114/114	Nucleus	7
Yue et al 2015 [20]	China	Breast cancer	160	51.0	All female	I–III	0–144	57/103	Nucleus	8
Zeng et al 2014 [21]	Japan	Liver cancer	144	62.7 ± 1.9	110/34	I–IV	NA	101/43	Nucleus	7

No.: number; y: year; m: month; NA: not available; NOS: Newcastle-Ottawa Scale; SG: Singapore Cohort; HK: Hong Kong Cohort.

Among all cohorts on ZNF217 expression, breast carcinoma was the most frequent cancer type (*n* = 4). Meanwhile, China (*n* = 3) became the major source region of literatures, followed by USA (*n* = 2) and France (*n* = 2). The median sample-size was 84, with a wide range from 44 to 319 (Table 2).

**Table 2:** Demographic information for included studies with ZNF217 expression

Reference	Country	Cancer type	No.	Mean age (y)	Male/Female	TNM stage	Median follow-up (m)	ZNF217 (−/+)	Subcellular localization	NOS score
Frietze et al 2014 [22]	USA	Breast cancer	319	NA	All female	I–IV	NA	160/159	Nucleus	7
Li et al 2014 [23]	China	Ovarian cancer	44	48.9	All female	I–IV	60	18/26	Nucleus	8
Li et al 2015 [24]	China	Colon cancer	82	57.4	42/40	I–IV	NA	27/55	Cytoplasm	7
Littlepage et al 2012 [25]	USA	Breast cancer	118	NA	All female	I–IV	0–144	59/59	Nucleus	7
Mao et al 2011 [26]	China	Glioblastoma	84	49.6 ± 5.7	46/38	II–III	NA	62/22	Nucleus	7
Nguyen et al 2014 [27]	France	Breast cancer	97	48.5	All female	NA	84	45/52	Nucleus and cytoplasm	8
Rahman et al 2012 [28]	Japan	Ovarian cancer	60	54.0	All female	I–IV	NA	40/20	Nucleus	6
Rooney et al 2004 [29]	UK	Colon cancer	100	69.0	56/44	I–III	54 (1–96)	43/57	Nucleus	7
Vendrell et al 2012 [30]	France	Breast cancer	78	55.8 ± 7.6	All female	I–IV	87 (2–169)	32/46	Nucleus	8

No.: number; y: year; m: month; NA: not available; NOS: Newcastle-Ottawa Scale.

### SALL4 levels and survival outcomes

#### 3-year overall survival

**Figure 2:**
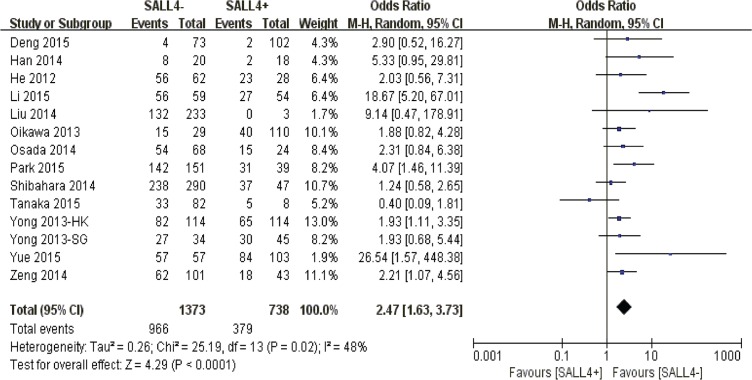
The correlation between SALL4 expression and 3-year overall survival in solid malignancies

The merged outcome indicated that a worse 3-year overall survival was obtained among patients with overexpression of SALL4 (*P* < 0.0001) (Figure 2).

#### 5-year overall survival

**Figure 3:**
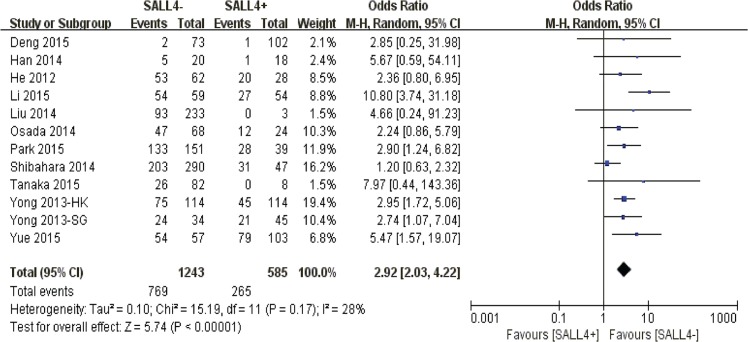
The correlation between SALL4 expression and 5-year overall survival in solid malignancies

Aberrant SALL4 positivity in nucleus was significantly correlated to the reduction of 5-year overall survival rate among solid cancer sufferers (*P* < 0.00001) (Figure 3).

#### 10-year overall survival

**Figure 4:**
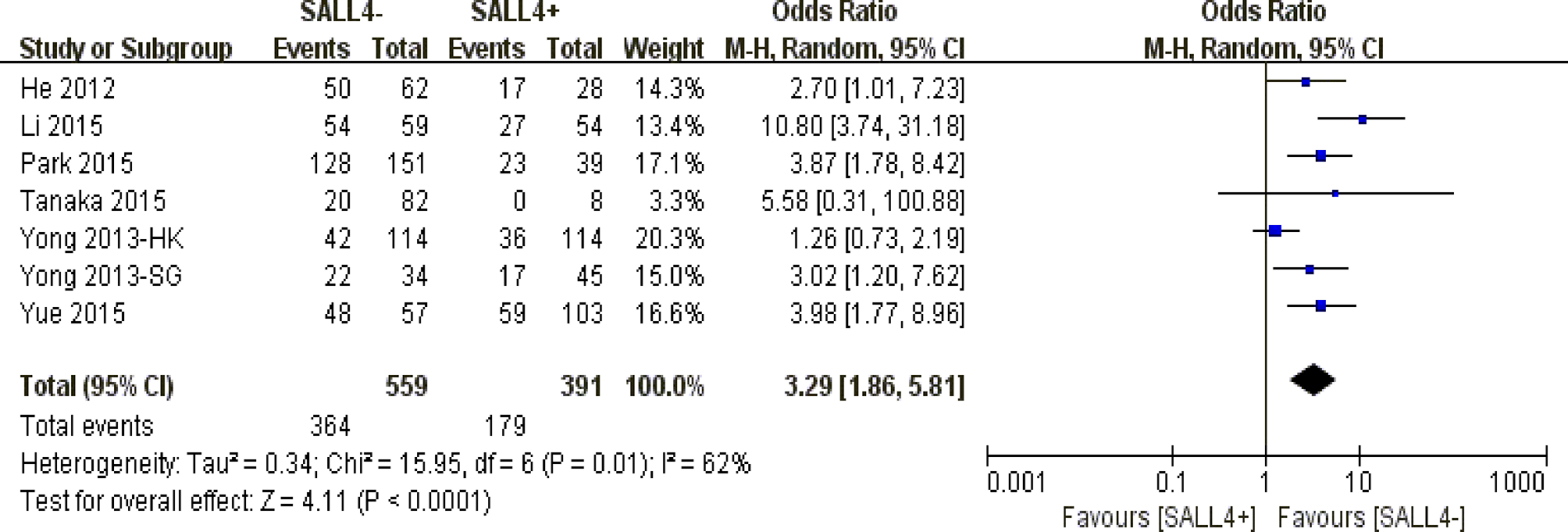
The correlation between SALL4 expression and 10-year overall survival in solid malignancies

Our pooled results discovered that abnormal SALL4 immunoreactivity played an unfavorable role on 10-year overall survival in solid malignancies (*P* < 0.0001) (Figure 4).

#### Disease free survival

**Figure 5:**
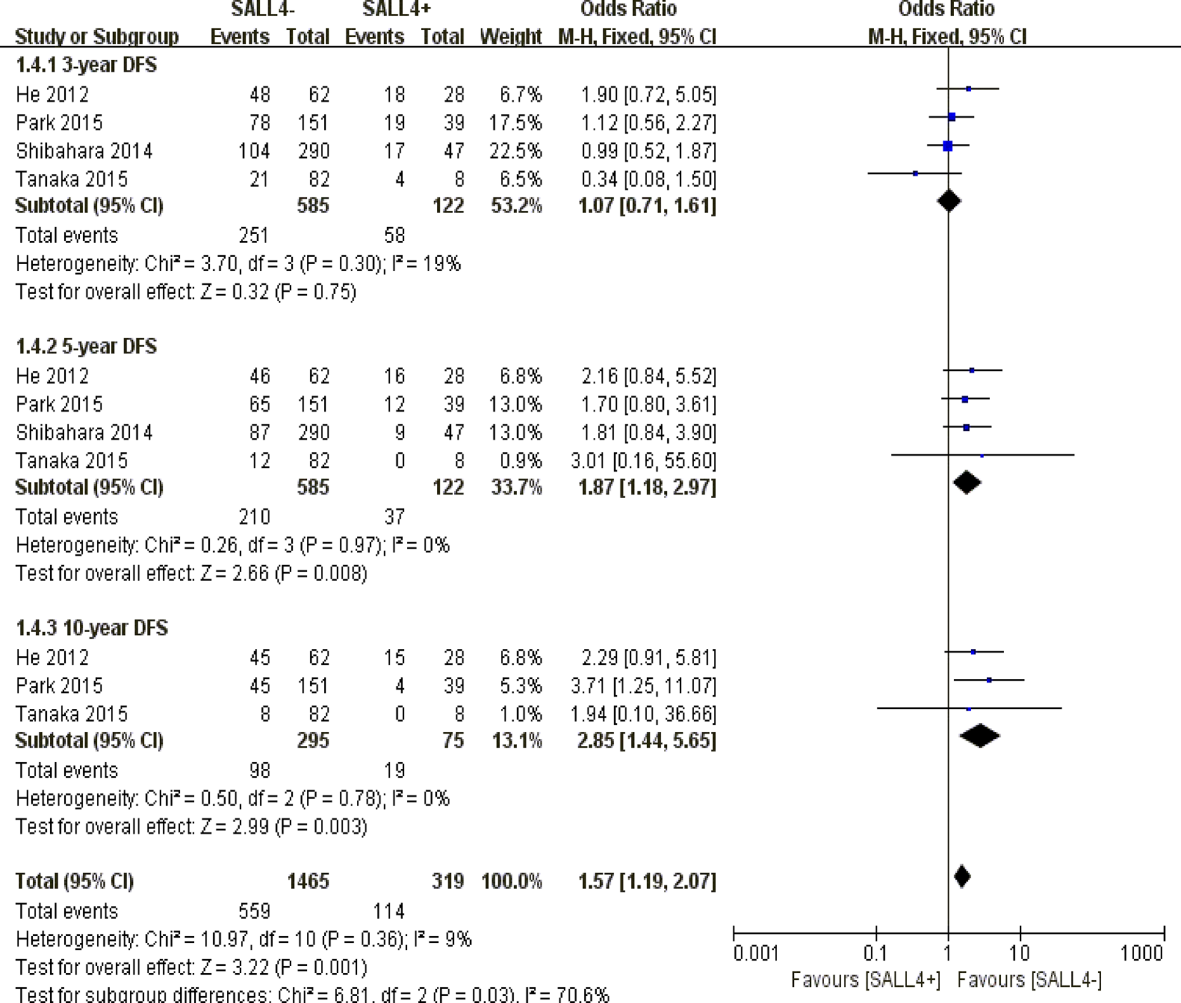
The correlation between SALL4 expression and disease-free survival in solid malignancies

With respect to 3-year disease free survival, there was no obvious correlation between SALL4 overexpression and prognostic expectancy in solid malignancies (*P* = 0.75). However, a poorer prognosis of cancer patients was observed concerning 5-year (*P* = 0.008) and 10-year disease free survival (*P* = 0.003) (Figure 5).

#### Subgroup analysis-cancer type

Original cohorts were subdivided into liver cancer and other cancer types respectively. Regarding 3-year follow-up duration, SALL4 redundancy was negatively related to overall prognosis in both subgroups (Liver cancer: *P* = 0.02) (Other types: *P* = 0.01). Meanwhile, its unfavorable impact on overall survival was similarly observed among patients with 5-year follow-up duration (Liver cancer: *P* < 0.00001) (Other types: *P* = 0.0003) (Supplementary Figure S1).

#### Subgroup analysis-mean-age

Attributed to aberrant SALL4 immunoreactivity, both subgroups (mean-age > 60 and mean-age < 60) disclosed a prognostic disadvantage among solid cancer patients, irrespective of 3-year (Mean-age > 60: *P* = 0.004) (Mean-age < 60: *P* < 0.0001) and 5-year overall survival (Mean-age > 60: *P* = 0.04) (Mean-age < 60: *P* < 0.00001) (Supplementary Figure S2).

#### Subgroup analysis-source region

All included studies were classified into Asian or non-Asian origin. As for Asian cohorts, excessive SALL4 expression was a negative indicator for 3-year (*P* < 0.00001) and 5-year survival expectancy (*P* < 0.00001). Similar outcomes were obtained in non-Asian subgroup (3-year: *P* < 0.00001) (5-year: *P* < 0.00001) (Supplementary Figure S3).

#### Subgroup analysis-sex predominance

No matter what gender preponderance it was, nuclear SALL4 staining was significantly correlated to poorer 3-year (Male predominance: *P* < 0.0001) (Female predominance: *P* = 0.01) and 5-year overall prognosis (Male predominance: *P* < 0.00001) (Female predominance: *P* = 0.0003) (Supplementary Figure S4).

### ZNF217 levels and survival outcomes

#### 3-year overall survival

**Figure 6:**
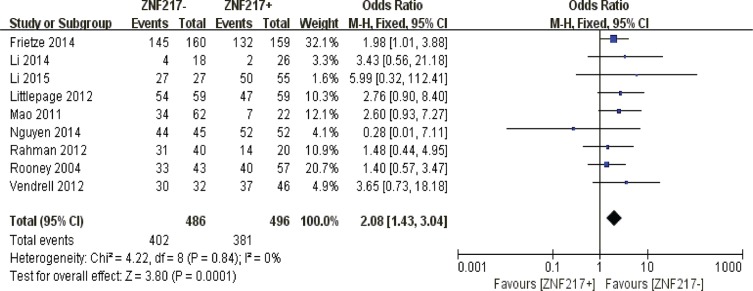
The correlation between ZNF217 expression and 3-year overall survival in solid malignancies

Redundant ZNF217 immunostaining was an unfavorable predictor of 3-year overall survival in solid malignancies (*P* = 0.0001) (Figure 6).

#### 5-year overall survival

**Figure 7:**
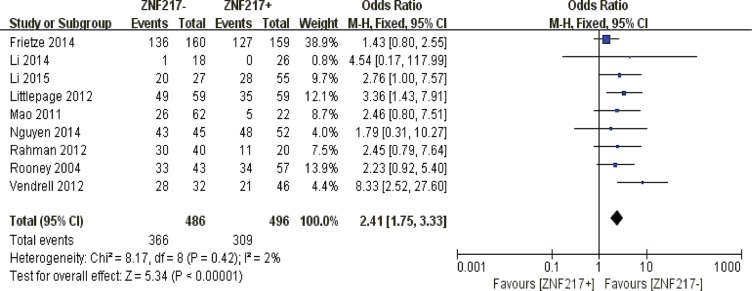
The correlation between ZNF217 expression and 5-year overall survival in solid malignancies

Our quantitative analysis suggested that ZNF217 overexpression in solid tumors exerted negative influences on 5-year overall survival (*P* < 0.00001) (Figure 7).

#### 10-year overall survival

**Figure 8:**
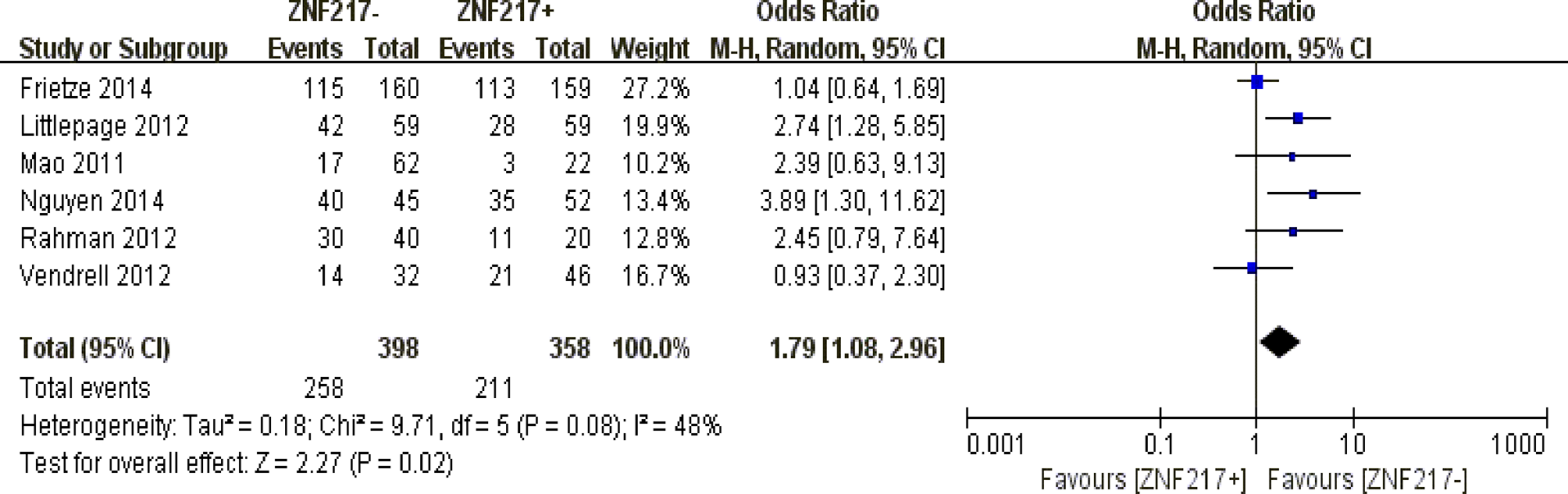
The correlation between ZNF217 expression and 10-year overall survival in solid malignancies

Excessive ZNF217 positivity in solid malignancies implied a significantly worse 10-year overall survival (*P* = 0.02) (Figure 8).

#### Disease free survival

**Figure 9:**
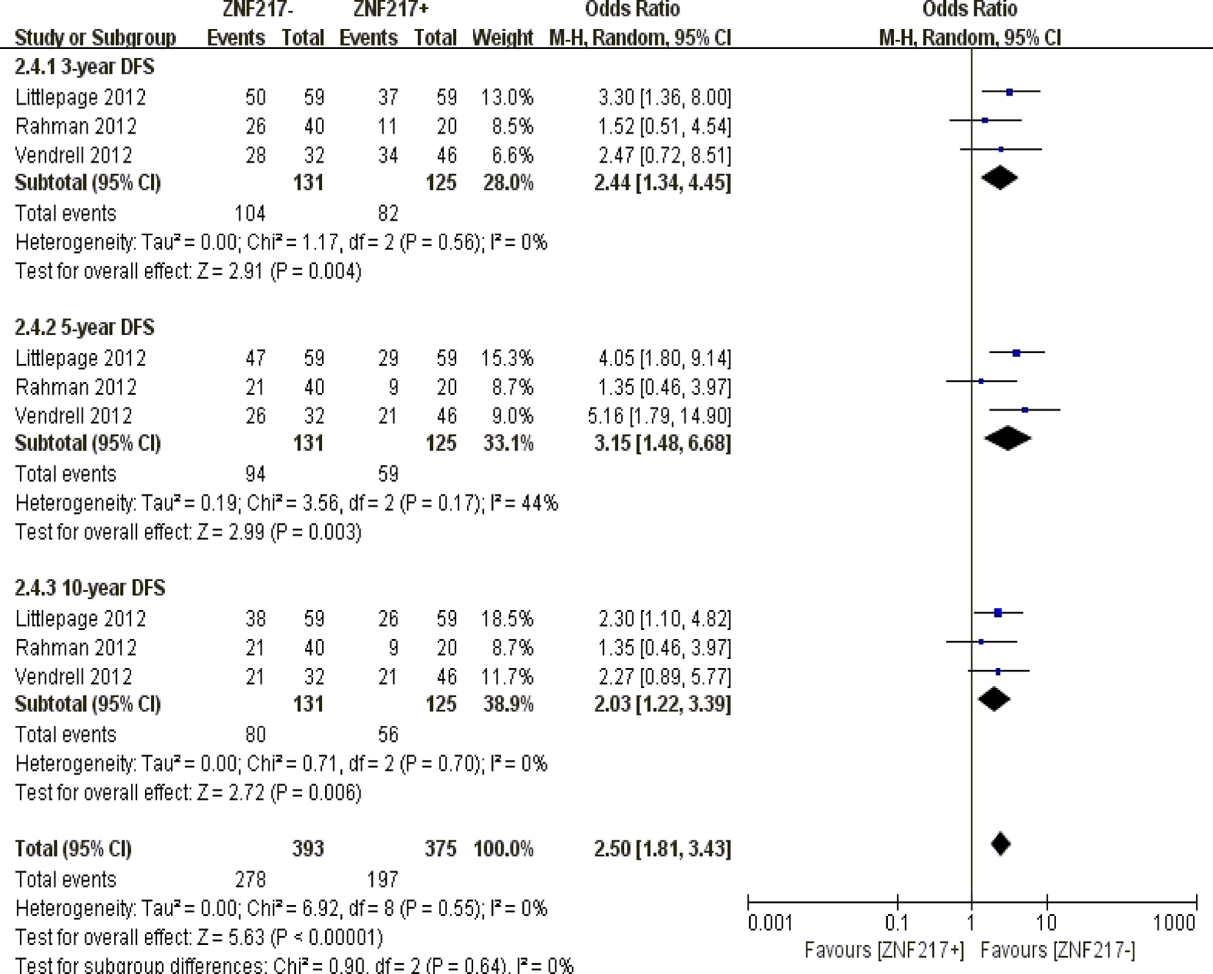
The correlation between ZNF217 expression and disease-free survival in solid malignancies

ZNF217 overexpression was negatively correlated with 3-year (*P* = 0.004), 5-year (*P* = 0.003) and 10-year disease frees survival (*P* = 0.006) (Figure 9).

#### Subgroup analysis-cancer type

Irrespective of cancer types, abnormal ZNF217 immunoreactivity was an indicator of worse 3-year (Breast cancer: *P* = 0.005) (Others types: *P* = 0.02) and 5-year prognosis respectively (Breast cancer: *P* = 0.01) (Others types: *P* = 0.0004) (Supplementary Figure S5).

#### Subgroup analysis-mean-age

ZNF217 redundancy was significantly linked to unfavorable prognosis among patients with mean-age > 50, irrespective of 3-year (*P* = 0.04) and 5-year follow-up duration (*P* < 0.00001). Nevertheless, there was no significant correlation between ZNF217 overexpression and long-term prognosis among patients with mean-age < 50 (3-year:*P* = 0.06) (5-year: *P* = 0.06) (Supplementary Figure S6).

#### Subgroup analysis-source region

It was statistically confirmed that ZNF217 redundancy was a negative indicator of 3-year (Asian: *P* = 0.02) (Non-Asian: *P* = 0.005) and 5-year overall survival in solid malignancies (Asian: *P* = 0.002) (Non-Asian: *P* = 0.002) (Supplementary Figure S7).

#### Subgroup analysis-sex predominance

Overexpression of ZNF217 indicated a worse 3-year (Male predominance: *P* = 0.03) (Female predominance: *P* = 0.002) and 5-year overall survival in solid malignancies (Male predominance: *P* = 0.002) (Female predominance: *P* < 0.0001) (Supplementary Figure S8).

#### Sensitivity analysis

Firstly, we performed sensitivity analysis by elimination of low-quality trials (NOS = 6). Outcomes remained stable in terms of 3-year (*P* < 0.0001) and 5-year (*P* < 0.00001) overall survival rate, despite that Han et al. [10] was excluded from pooled analysis of SALL4 expression. Similarly, pooled outcomes of ZNF217 maintained stablealthough Rahman et al. [28] was removed, regardless of 3-year (*P* = 0.0002) and 5-year (*P* < 0.00001) overall survival in solid malignancies.

Secondly, we implemented another sensitivity analysis by excluding studies with cytoplasmic staining. Nguyen et al. [27] and Li et al. [24] were accordingly eliminated from the meta-analysis of ZNF217. Stable results were confirmed in terms of 3-year (*P* = 0.0002) or 5-year overall survival (*P* < 0.00001).

#### Publication bias

**Figure 10:**
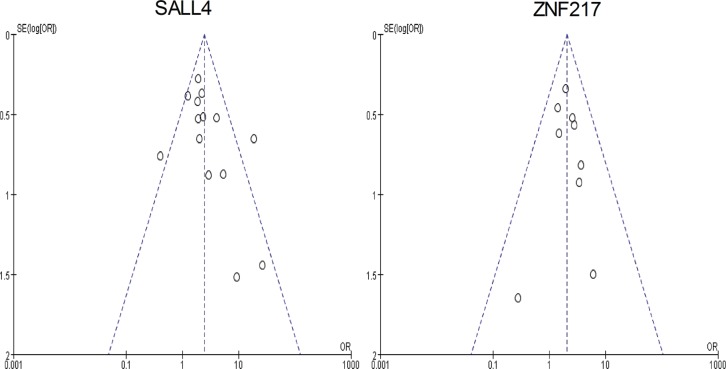
The funnel plots of this meta-analysis

The funnel plots of 3-year overall survival in SALL4 and ZNF217 group were both graphically symmetric. Additionally, Egger's test (SALL4: *P* = 0.125; ZNF217: *P* = 0.790) and Begg's test (SALL4: *P* = 0.080; ZNF217: *P* = 0.466) jointly confirmed that there was no publication bias among the included studies in both groups (Figure 10).

## DISCUSSION

As is widely proposed, cancer cells share similar biological behavior with progenitor cells. Both types of cell display undifferentiated phenotypes and retain immortal states [31]. Therefore, oncofetal proteins have great potentials to become therapeutic target spots.

SALL4 encodes a zinc-finger transcription factor which functionally interacts with cellular effectors to activate the early-stage embryogenesis [32, 33]. Researchers have experimentally confirmed the carcinogenic role of SALL4 in a variety of malignancies. Normally, its expression is dramatically shrunk within mature tissues. The abnormal reemergence of SALL4 has been closely linked to neoplastic transformation in susceptible crowds [34]. Individual cohorts have sporadically revealed the unfavorably prognostic role of SALL4 in certain types of cancer. However, a comprehensive conclusion remains in scarcity. Our study is the first meta-analysis to conclude that SALL4 redundancy is a negative indicator towards long-term survival expectancy in solid malignancies, irrespective of cancer types or source regions. It is academically acknowledged that metastatic dissemination contributes to more than 90 percent of cancer mortality [35]. In-depth investigations have molecularly demonstrated that SALL4 serves as a core activator to elevate the expression of downstream target genes such as Bmi-1 and c-Myc, which subsequently trigger epithelial mesenchymal transition or angiogenesis [36, 37]. This is a probable explanation of the worse prognosis with SALL4 overexpression. Additionally, chemo-resistance is another challenge for therapeutic efficacies. It is already known that upregulation of c-Myc and ABCG2 exacerbates the severity of chemical resistance in multiple cancers. Overexpression of SALL4 is able to enhance the phenotypic positivity of both molecules, therefore its prognostic role is fairly comprehensible [12, 14]. On the other hand, it seems that Li et al. and Tanaka et al. are responsible for the majority of internal heterogeneity across studies, since the removal of both trials thoroughly wipe off the *I*^2^ value from 48% to 0%. Contradictory to his own hypothesis, Tanaka et al. [18] (accounting for 13% of heterogeneity) attributed the opposite outcome in SALL4 positive group to the unbalanced patient distribution (SALL4-negative group consists of more intrahepatic cholangiocarcinoma patients who feature a lower SALL4 staining percentage but a poorer prognosis). Nevertheless, Deng et al. [9] described a comparable SALL4 phenotypic pattern in intrahepatic cholangiocarcinoma against hepatocellular carcinoma participants, as well as the similar prognostic expectancy. On this account, more relevant studies are still necessary for further subgroup analysis. Moreover, the partial contribution of Li et al. [12] (accounting for 35% of heterogeneity) in heterogeneity mainly blames on the broader gap between different SALL4 expression levels regarding survival prognosis.

ZNF217 is a newly cloned gene with oncogenic properties. It encodes a Kruppel-like zinc-finger transcription factor which takes effect by nuclear interplay with associated molecules. This transcriptional activator was initially correlated to breast cancer in 1998, capable of promoting immortalization and restoring embryonic phenotypes [38]. It is well characterized that ZNF217-HER3-AKT pathway is frequently activated in invasive ductal breast cancer, which secondarily induces a more aggressive behavior and drug insensitivity through phenotypic alteration [25]. Meanwhile, following overexpression of ZNF217, oncogene PyMT is abnormally amplified and switches the epithelial phenotype towards myomesenchymal-like cell differentiation in murine models, indicating a higher risk of dissemination. C-Myc, a neoplastic effector, functions as a downstream target of ZNF217. A considerable amount of malignant spreads as well as chemical refractoriness arises from the overexpression of c-Myc. As a consequence, it seems theoretically rational that ZNF217 is a predicator of shorter survival in solid malignancies [25, 39]. In agreement with previous individual studies, we meta-analyzed all relevant cohorts and made a globally first statement that ZNF217 amplification had an implication of worse prognosis in solid malignancies, despite of cancer subtypes or sex predominance. Interestingly, apart from the regular staining in nuclei, the discovery by Li et al. [24] suggests a comparably indicative role of ZNF217 cytoplasmic immunoreactivity. This exception of subcellular localization reminds us that further incisive evidence is still required.

There are some limitations in our meta-analysis that should be considered in interpreting the outcomes. Firstly, our pooled analysis is fully on the strength of observational cohorts, which may partially lead to selection bias despite the baseline characteristics are comparable. Secondly, in spite of a thorough database searching, the total amount of included participants may still be insufficient to draw a consolidated result.

Taken together, we made a statistical evidence of unfavorable role of SALL4 and ZNF217 overexpression on survival expectancy. Their pharmaceutical blockage may benefit distant recurrence or chemical resistance.

## MATERIALS AND METHODS

### Literature search

We carried out the literature retrieval by searching databases of PubMed and Web of Science until October 2015. The search terms “SALL4 or ZNF217” was utilized to amplify the searching range. In order to guarantee the accuracy and completeness, both full-texts and citation list of potential studies were examined, apart from the abstract screening.

### Study selection

Studies that accorded with the following criteria were included: 1. English written articles; 2. Human studies regarding correlation between SALL4 (or ZNF217) expression and clinical prognosis in solid malignancies; 3. The expression of SALL4 or ZNF217 was detected by immunohistochemistry (IHC).

Studies were ruled out due to the following reasons: 1. Inadequate survival data for further quantification or the follow-up duration was shorter than 3 years; 2. Overlapped or duplicated studies; 3. Inappropriate article types such as reviews and case-reports; 4. Studies involving less than 10 participants as its sample-size.

Two investigators independently implemented the selection process and any disagreement was settled by mutual discussion.

### Data extraction

Standardized extraction forms were applied for data extraction. Concerning the classification standards of SALL4 and ZNF217 expression, we generally recognized low-expression as < 25% of cell positivity while high-expression as > 25% of cell positivity. However, this criterion was adaptively adjusted if necessary, mainly based on original subgroups within individual trials. Two authors collaboratively extracted prognostic materials from main texts or Kaplan-Meier curves.

### Methodological assessment

Owing to the observational properties of included studies, a Newcastle-Ottawa Scale (NOS) was employed for methodological evaluation. The scale consisted of three categories including selection, comparability and outcome, with a maximum score of nine. Studies graded with more than six scores were identified as high quality trials in methodology. Each study was appraised by two evaluators in an independent way. Any disapproval was resolved by mutual discussion.

### Statistical analysis

Our quantitative calculation was performed on Review Manager 5.3 under Cochrane Collaboration protocols. Odds ratio (OR) along with Mantel-Haenszel model were adopted for dichotomous variables. We acknowledged *I*^2^ as a heterogeneity indicator with its value < 25%, 25%–50% and > 50% defined as low, moderate and significant heterogeneity respectively. A moderate or significant heterogeneity was adjusted by a random-effects model, otherwise a fix-effects model was preferred. The statistical significance within all comparisons was mathematically signified as *P* < 0.05. Moreover, sensitivity analysis was applied to examine the stability of pooled outcomes. Publication bias was statistically analyzed via Egger's test and Begg's test, based on the results from STATA 12.0.

## SUPPLEMENTARY MATERIALS FIGURES


