# Supplementary material for: Oncogenic protein SALL4 and ZNF217 as prognostic indicators in solid cancers: a meta-analysis of individual studies
Source: Oncotarget. 2016 Mar 21;7(17):24314–25. doi: 10.18632/oncotarget.8237 (PMC5029703; doi:10.18632/oncotarget.8237)
Supplement: Supplementary file 1 [file oncotarget-07-24314-s001.pdf]

Oncogenic protein SALL4 and ZNF217 as prognostic indicators in solid cancers: a meta-analysis of individual studies

Supplementary Materials

Study or Subgroup	SALL4- Events	SALL4- Total	SALL4+ Events	SALL4+ Total	Weight	Odds Ratio M-H, Random, 95% CI
**1.5.1 Liver cancer of 3-year OS**						
Deng 2015	4	73	2	102	2.5%	2.90 [0.52, 16.27]
Han 2014	8	20	2	18	2.5%	5.33 [0.95, 29.81]
Liu 2014	132	233	0	3	1.1%	9.14 [0.47, 178.91]
Oikawa 2013	15	29	40	110	5.2%	1.88 [0.82, 4.28]
Park 2015	142	151	31	39	4.4%	4.07 [1.46, 11.39]
Shibahara 2014	238	290	37	47	5.5%	1.24 [0.58, 2.65]
Tanaka 2015	33	82	30	45	5.5%	0.34 [0.16, 0.72]
Yong 2013-HK	82	114	65	114	6.4%	1.93 [1.11, 3.35]
Yong 2013-SG	27	34	30	45	4.4%	1.93 [0.68, 5.44]
Zeng 2014	62	101	18	43	5.6%	2.21 [1.07, 4.56]
**Subtotal (95% CI)**		**1127**		**566**	**42.9%**	**1.81 [1.09, 3.01]**
Total events	743		255			
Heterogeneity: Tau² = 0.38; Chi² = 24.85, df = 9 (P = 0.003); I² = 64%						
Test for overall effect: Z = 2.28 (P = 0.02)						
**1.5.2 Other types of 3-year OS**						
He 2012	56	62	23	28	3.6%	2.03 [0.56, 7.31]
Li 2015	56	59	27	54	3.6%	18.67 [5.20, 67.01]
Osada 2014	54	68	15	24	4.5%	2.31 [0.84, 6.38]
Yue 2015	57	57	84	103	1.2%	26.54 [1.57, 448.38]
**Subtotal (95% CI)**		**246**		**209**	**12.7%**	**5.57 [1.50, 20.67]**
Total events	223		149			
Heterogeneity: Tau² = 1.19; Chi² = 10.22, df = 3 (P = 0.02); I² = 71%						
Test for overall effect: Z = 2.57 (P = 0.01)						
**1.5.3 Liver cancer of 5-year OS**						
Deng 2015	2	73	1	102	1.5%	2.85 [0.25, 31.98]
Han 2014	5	20	1	18	1.7%	5.67 [0.59, 54.11]
Liu 2014	93	233	0	3	1.1%	4.66 [0.24, 91.23]
Park 2015	133	151	28	39	5.1%	2.90 [1.24, 6.82]
Shibahara 2014	203	290	31	47	5.9%	1.20 [0.63, 2.32]
Tanaka 2015	26	82	0	8	1.1%	7.97 [0.44, 143.36]
Yong 2013-HK	75	114	45	114	6.4%	2.95 [1.72, 5.06]
Yong 2013-SG	24	34	21	45	4.7%	2.74 [1.07, 7.04]
**Subtotal (95% CI)**		**997**		**376**	**27.5%**	**2.38 [1.70, 3.33]**
Total events	561		127			
Heterogeneity: Tau² = 0.00; Chi² = 6.55, df = 7 (P = 0.48); I² = 0%						
Test for overall effect: Z = 5.06 (P < 0.00001)						
**1.5.4 Other types of 5-year OS**						
He 2012	53	62	20	28	4.2%	2.36 [0.80, 6.95]
Li 2015	54	59	27	54	4.3%	10.80 [3.74, 31.18]
Osada 2014	47	68	12	24	4.7%	2.24 [0.86, 5.79]
Yue 2015	54	57	79	103	3.7%	5.47 [1.57, 19.07]
**Subtotal (95% CI)**		**246**		**209**	**16.9%**	**4.10 [1.91, 8.81]**
Total events	208		138			
Heterogeneity: Tau² = 0.31; Chi² = 6.04, df = 3 (P = 0.11); I² = 50%						
Test for overall effect: Z = 3.61 (P = 0.0003)						
**Total (95% CI)**		**2616**		**1360**	**100.0%**	**2.62 [1.88, 3.64]**
Total events	1735		669			
Heterogeneity: Tau² = 0.37; Chi² = 60.96, df = 25 (P < 0.0001); I² = 59%						
Test for overall effect: Z = 5.73 (P < 0.00001)						
Test for subgroup differences: Chi² = 4.63, df = 3 (P = 0.20), I² = 35.3%						

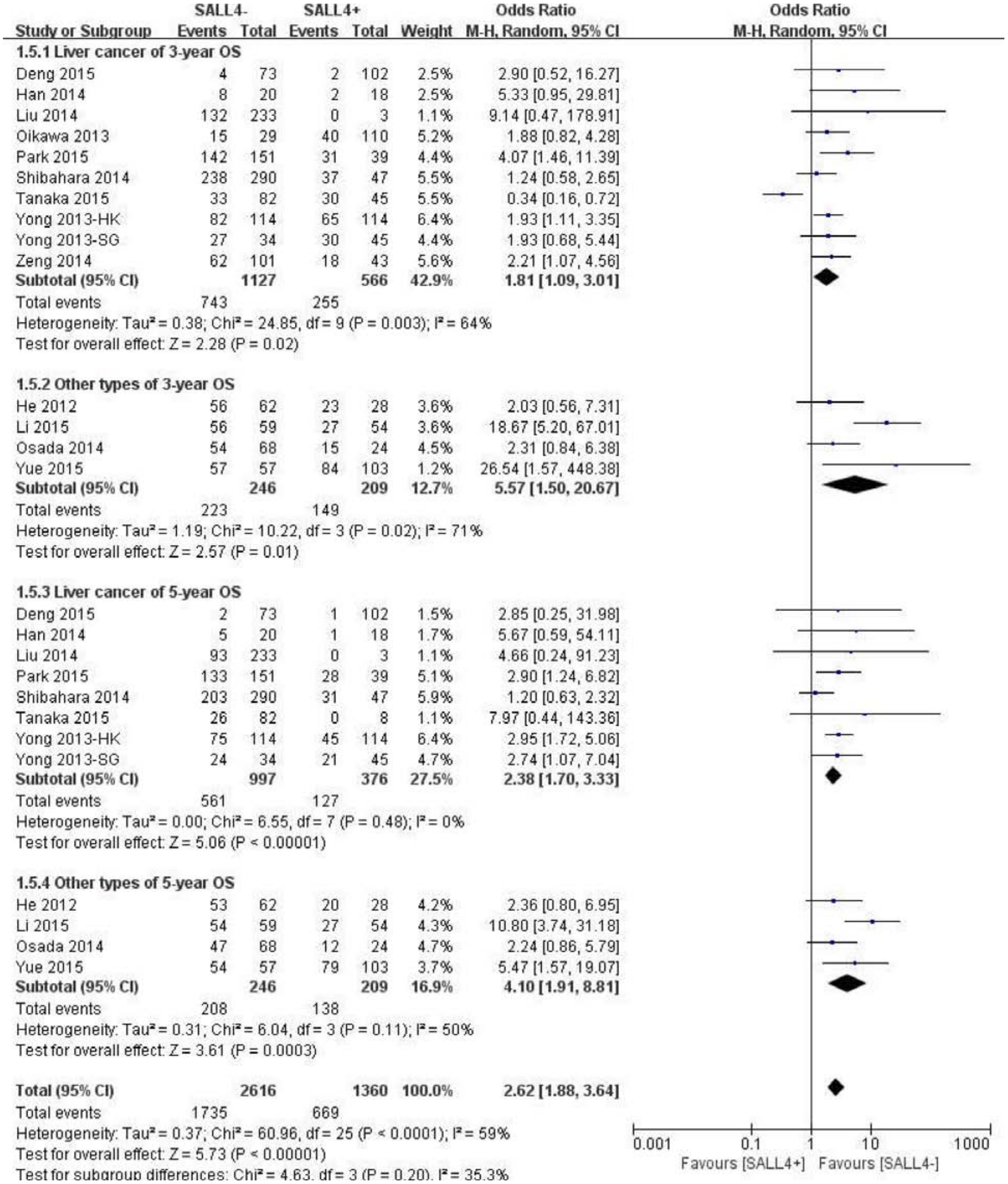

Supplementary Figure S1: The correlation between SALL4 expression and prognosis based on different cancer types.

Study or Subgroup	SALL4- Events	SALL4- Total	SALL4+ Events	SALL4+ Total	Weight	Odds Ratio M-H, Random, 95% CI
**1.6.1 Mean-age>60 of 3-year OS**						
He 2012	56	62	23	28	3.2%	2.03 [0.56, 7.31]
Liu 2014	132	233	0	3	0.7%	9.14 [0.47, 178.91]
Osada 2014	54	68	15	24	4.6%	2.31 [0.84, 6.38]
Shibahara 2014	238	290	37	47	6.4%	1.24 [0.58, 2.65]
Zeng 2014	62	101	18	43	6.8%	2.21 [1.07, 4.56]
**Subtotal (95% CI)**		**754**		**145**	**21.7%**	**1.88 [1.22, 2.90]**
Total events	542		93			
Heterogeneity: Tau² = 0.00; Chi² = 2.62, df = 4 (P = 0.62); I² = 0%						
Test for overall effect: Z = 2.86 (P = 0.004)						
**1.6.2 Mean-age<60 of 3-year OS**						
Deng 2015	4	73	2	102	2.0%	2.90 [0.52, 16.27]
Han 2014	8	20	2	18	2.0%	5.33 [0.95, 29.81]
Li 2015	56	59	27	54	3.3%	18.67 [5.20, 67.01]
Oikawa 2013	15	29	40	110	5.9%	1.88 [0.82, 4.28]
Park 2015	142	151	31	39	4.5%	4.07 [1.46, 11.39]
Yong 2013-HK	82	114	65	114	8.6%	1.93 [1.11, 3.35]
Yong 2013-SG	27	34	30	45	4.4%	1.93 [0.68, 5.44]
Yue 2015	57	57	84	103	0.8%	26.54 [1.57, 448.38]
**Subtotal (95% CI)**		**537**		**585**	**31.5%**	**3.49 [1.91, 6.38]**
Total events	391		281			
Heterogeneity: Tau² = 0.37; Chi² = 15.67, df = 7 (P = 0.03); I² = 55%						
Test for overall effect: Z = 4.07 (P < 0.0001)						
**1.6.3 Mean-age>60 of 5-year OS**						
He 2012	53	62	20	28	4.2%	2.36 [0.80, 6.95]
Liu 2014	93	233	0	3	0.7%	4.66 [0.24, 91.23]
Osada 2014	47	68	12	24	4.9%	2.24 [0.86, 5.79]
Shibahara 2014	203	290	31	47	7.5%	1.20 [0.63, 2.32]
**Subtotal (95% CI)**		**653**		**102**	**17.3%**	**1.66 [1.03, 2.67]**
Total events	396		63			
Heterogeneity: Tau² = 0.00; Chi² = 2.17, df = 3 (P = 0.54); I² = 0%						
Test for overall effect: Z = 2.08 (P = 0.04)						
**1.6.4 Mean-age<60 of 5-year OS**						
Deng 2015	2	73	1	102	1.1%	2.85 [0.25, 31.98]
Han 2014	5	20	1	18	1.2%	5.67 [0.59, 54.11]
Li 2015	54	59	27	54	4.3%	10.80 [3.74, 31.18]
Park 2015	133	151	28	39	5.7%	2.90 [1.24, 6.82]
Yong 2013-HK	75	114	45	114	8.8%	2.95 [1.72, 5.06]
Yong 2013-SG	24	34	21	45	5.0%	2.74 [1.07, 7.04]
Yue 2015	54	57	79	103	3.4%	5.47 [1.57, 19.07]
**Subtotal (95% CI)**		**508**		**475**	**29.4%**	**3.60 [2.52, 5.15]**
Total events	347		202			
Heterogeneity: Tau² = 0.00; Chi² = 5.90, df = 6 (P = 0.43); I² = 0%						
Test for overall effect: Z = 7.03 (P < 0.00001)						
**Total (95% CI)**		**2452**		**1307**	**100.0%**	**2.74 [2.10, 3.57]**
Total events	1676		639			
Heterogeneity: Tau² = 0.13; Chi² = 35.07, df = 23 (P = 0.05); I² = 34%						
Test for overall effect: Z = 7.46 (P < 0.00001)						
Test for subgroup differences: Chi² = 9.67, df = 3 (P = 0.02), I² = 69.0%						

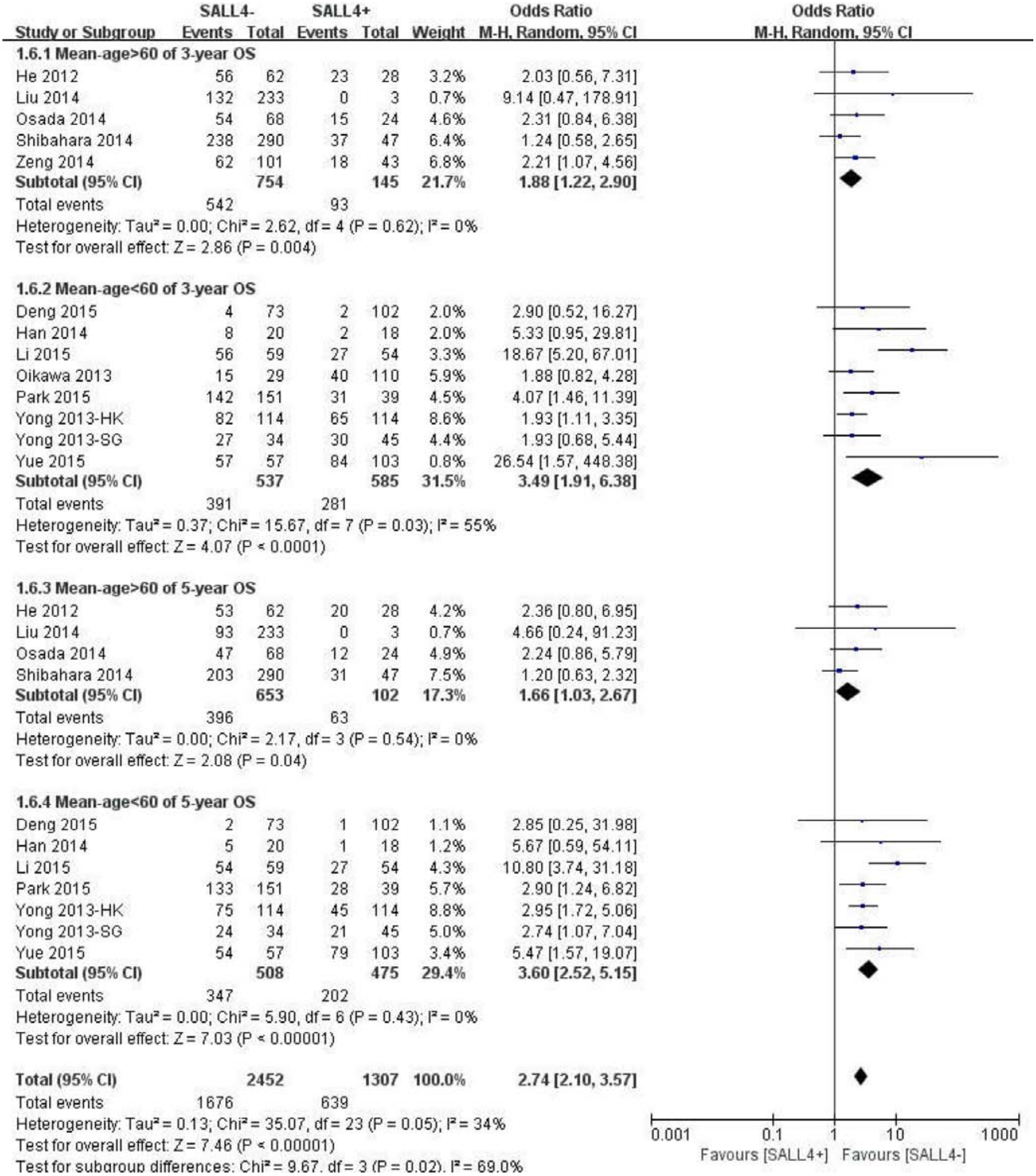

Supplementary Figure S2: The correlation between SALL4 expression and prognosis based on different mean-age.

Study or Subgroup	SALL4- Events	SALL4- Total	SALL4+ Events	SALL4+ Total	Weight	Odds Ratio M-H, Random, 95% CI
**1.7.1 Asian studies of 3-year OS**						
Deng 2015	4	73	2	102	2.1%	2.90 [0.52, 16.27]
Han 2014	8	20	2	18	2.1%	5.33 [0.95, 29.81]
He 2012	56	62	23	28	3.3%	2.03 [0.56, 7.31]
Oikawa 2013	15	29	40	110	5.6%	1.88 [0.82, 4.28]
Osada 2014	54	68	15	24	4.5%	2.31 [0.84, 6.38]
Park 2015	142	151	31	39	4.4%	4.07 [1.46, 11.39]
Shibahara 2014	238	290	37	47	6.1%	1.24 [0.58, 2.65]
Tanaka 2015	33	82	5	8	2.6%	0.40 [0.09, 1.81]
Yong 2013-HK	82	114	65	114	7.8%	1.93 [1.11, 3.35]
Yong 2013-SG	27	34	30	45	4.4%	1.93 [0.68, 5.44]
Yue 2015	57	57	84	103	0.9%	26.54 [1.57, 448.38]
Zeng 2014	62	101	18	43	6.3%	2.21 [1.07, 4.56]
**Subtotal (95% CI)**		**1081**		**681**	**50.0%**	**2.02 [1.48, 2.75]**
Total events	778		352			
Heterogeneity: Tau² = 0.04; Chi² = 12.73, df = 11 (P = 0.31); I² = 14%						
Test for overall effect: Z = 4.46 (P < 0.00001)						
**1.7.2 Non-Asian studies of 3-year OS**						
Li 2015	56	59	27	54	3.3%	18.67 [5.20, 67.01]
Liu 2014	132	233	0	3	0.8%	9.14 [0.47, 178.91]
**Subtotal (95% CI)**		**292**		**57**	**4.1%**	**16.70 [5.16, 54.04]**
Total events	188		27			
Heterogeneity: Tau² = 0.00; Chi² = 0.19, df = 1 (P = 0.67); I² = 0%						
Test for overall effect: Z = 4.70 (P < 0.00001)						
**1.7.3 Asian studies of 5-year OS**						
Deng 2015	2	73	1	102	1.2%	2.85 [0.25, 31.98]
Han 2014	5	20	1	18	1.3%	5.67 [0.59, 54.11]
He 2012	53	62	20	28	4.1%	2.36 [0.80, 6.95]
Osada 2014	47	68	12	24	4.8%	2.24 [0.86, 5.79]
Park 2015	133	151	28	39	5.4%	2.90 [1.24, 6.82]
Shibahara 2014	203	290	31	47	6.9%	1.20 [0.63, 2.32]
Tanaka 2015	26	82	0	8	0.8%	7.97 [0.44, 143.36]
Yong 2013-HK	75	114	45	114	7.9%	2.95 [1.72, 5.06]
Yong 2013-SG	24	34	21	45	4.9%	2.74 [1.07, 7.04]
Yue 2015	54	57	79	103	3.4%	5.47 [1.57, 19.07]
**Subtotal (95% CI)**		**951**		**528**	**40.8%**	**2.46 [1.83, 3.31]**
Total events	622		238			
Heterogeneity: Tau² = 0.00; Chi² = 8.06, df = 9 (P = 0.53); I² = 0%						
Test for overall effect: Z = 5.95 (P < 0.00001)						
**1.7.4 Non-Asian studies of 5-year OS**						
Li 2015	54	59	27	54	4.2%	10.80 [3.74, 31.18]
Liu 2014	93	233	0	3	0.8%	4.66 [0.24, 91.23]
**Subtotal (95% CI)**		**292**		**57**	**5.0%**	**9.82 [3.62, 26.67]**
Total events	147		27			
Heterogeneity: Tau² = 0.00; Chi² = 0.27, df = 1 (P = 0.60); I² = 0%						
Test for overall effect: Z = 4.48 (P < 0.00001)						
**Total (95% CI)**		**2616**		**1323**	**100.0%**	**2.67 [2.02, 3.51]**
Total events	1735		644			
Heterogeneity: Tau² = 0.17; Chi² = 41.38, df = 25 (P = 0.02); I² = 40%						
Test for overall effect: Z = 6.97 (P < 0.00001)						
Test for subgroup differences: Chi² = 19.07, df = 3 (P = 0.0003), I² = 84.3%						

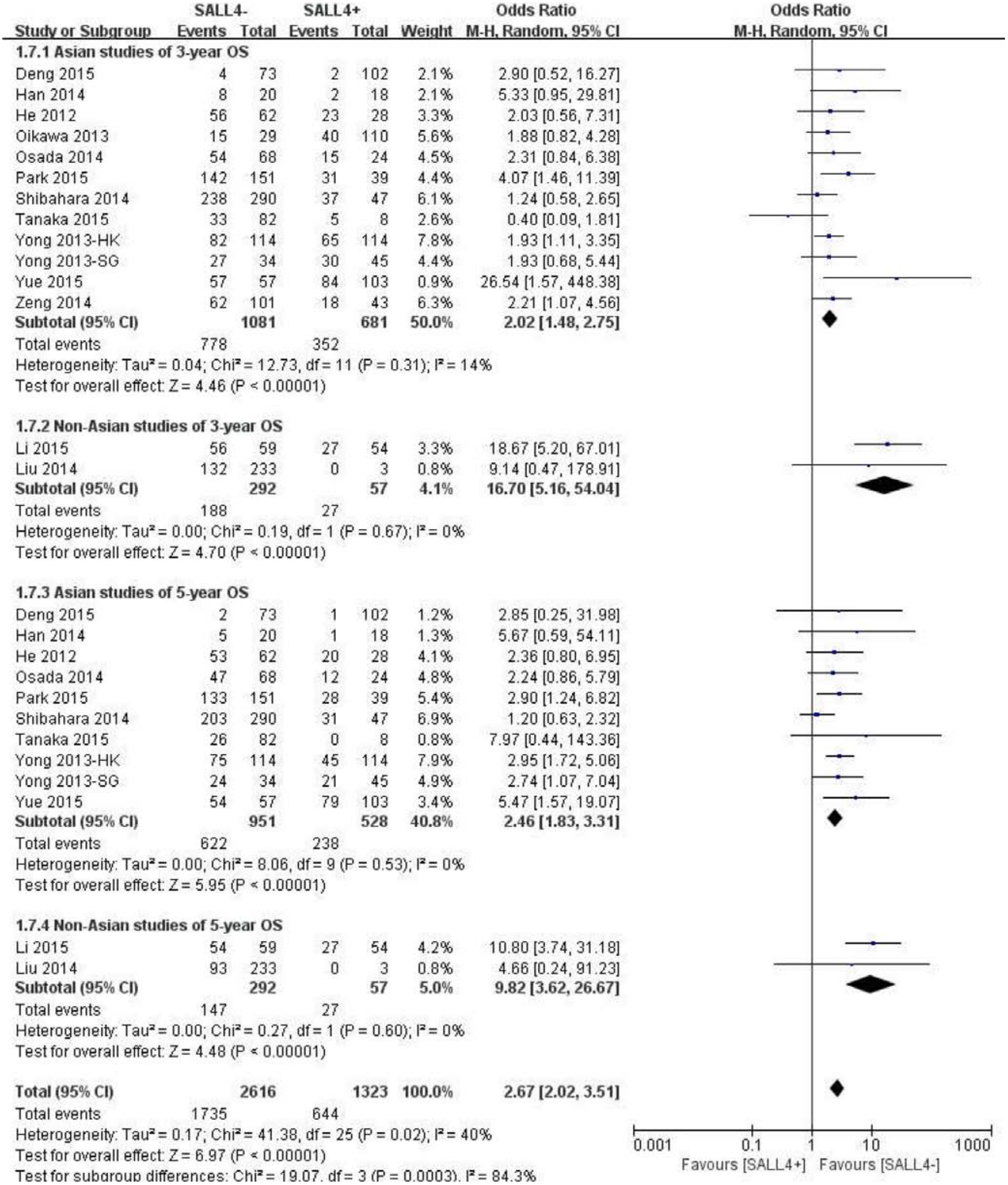

Supplementary Figure S3: The correlation between SALL4 expression and prognosis based on different source regions.

Study or Subgroup	SALL4- Events	SALL4- Total	SALL4+ Events	SALL4+ Total	Weight	Odds Ratio M-H, Random, 95% CI
**1.8.1 Male predominance of 3-year OS**						
Deng 2015	4	73	2	102	2.1%	2.90 [0.52, 16.27]
Han 2014	8	20	2	18	2.1%	5.33 [0.95, 29.81]
Liu 2014	132	233	0	3	0.8%	9.14 [0.47, 178.91]
Oikawa 2013	15	29	40	110	5.6%	1.88 [0.82, 4.28]
Park 2015	142	151	31	39	4.4%	4.07 [1.46, 11.39]
Shibahara 2014	238	290	37	47	6.1%	1.24 [0.58, 2.65]
Tanaka 2015	33	82	5	8	2.6%	0.40 [0.09, 1.81]
Yong 2013-HK	82	114	65	114	7.8%	1.93 [1.11, 3.35]
Yong 2013-SG	27	34	30	45	4.4%	1.93 [0.68, 5.44]
Zeng 2014	62	101	18	43	6.3%	2.21 [1.07, 4.56]
**Subtotal (95% CI)**		**1127**		**529**	**42.2%**	**1.96 [1.41, 2.72]**
Total events	743		230			
Heterogeneity: Tau² = 0.03; Chi² = 10.26, df = 9 (P = 0.33); I² = 12%						
Test for overall effect: Z = 4.02 (P < 0.0001)						
**1.8.2 Female predominance of 3-year OS**						
He 2012	56	62	23	28	3.3%	2.03 [0.56, 7.31]
Li 2015	56	59	27	54	3.3%	18.67 [5.20, 67.01]
Osada 2014	54	68	15	24	4.5%	2.31 [0.84, 6.38]
Yue 2015	57	57	84	103	0.9%	26.54 [1.57, 448.38]
**Subtotal (95% CI)**		**246**		**209**	**11.9%**	**5.57 [1.50, 20.67]**
Total events	223		149			
Heterogeneity: Tau² = 1.19; Chi² = 10.22, df = 3 (P = 0.02); I² = 71%						
Test for overall effect: Z = 2.57 (P = 0.01)						
**1.8.3 Male predominance of 5-year OS**						
Deng 2015	2	73	1	102	1.2%	2.85 [0.25, 31.98]
Han 2014	5	20	1	18	1.3%	5.67 [0.59, 54.11]
Liu 2014	93	233	0	3	0.8%	4.66 [0.24, 91.23]
Park 2015	133	151	28	39	5.4%	2.90 [1.24, 6.82]
Shibahara 2014	203	290	31	47	6.9%	1.20 [0.63, 2.32]
Tanaka 2015	26	82	0	8	0.8%	7.97 [0.44, 143.36]
Yong 2013-HK	75	114	45	114	7.9%	2.95 [1.72, 5.06]
Yong 2013-SG	24	34	21	45	4.9%	2.74 [1.07, 7.04]
**Subtotal (95% CI)**		**997**		**376**	**29.3%**	**2.38 [1.70, 3.33]**
Total events	561		127			
Heterogeneity: Tau² = 0.00; Chi² = 6.55, df = 7 (P = 0.48); I² = 0%						
Test for overall effect: Z = 5.06 (P < 0.00001)						
**1.8.4 Female predominance of 5-year OS**						
He 2012	53	62	20	28	4.1%	2.36 [0.80, 6.95]
Li 2015	54	59	27	54	4.2%	10.80 [3.74, 31.18]
Osada 2014	47	68	12	24	4.8%	2.24 [0.86, 5.79]
Yue 2015	54	57	79	103	3.4%	5.47 [1.57, 19.07]
**Subtotal (95% CI)**		**246**		**209**	**16.6%**	**4.10 [1.91, 8.81]**
Total events	208		138			
Heterogeneity: Tau² = 0.31; Chi² = 6.04, df = 3 (P = 0.11); I² = 50%						
Test for overall effect: Z = 3.61 (P = 0.0003)						
**Total (95% CI)**		**2616**		**1323**	**100.0%**	**2.67 [2.02, 3.51]**
Total events	1735		644			
Heterogeneity: Tau² = 0.17; Chi² = 41.38, df = 25 (P = 0.02); I² = 40%						
Test for overall effect: Z = 6.97 (P < 0.00001)						
Test for subgroup differences: Chi² = 4.87, df = 3 (P = 0.18), I² = 38.4%						

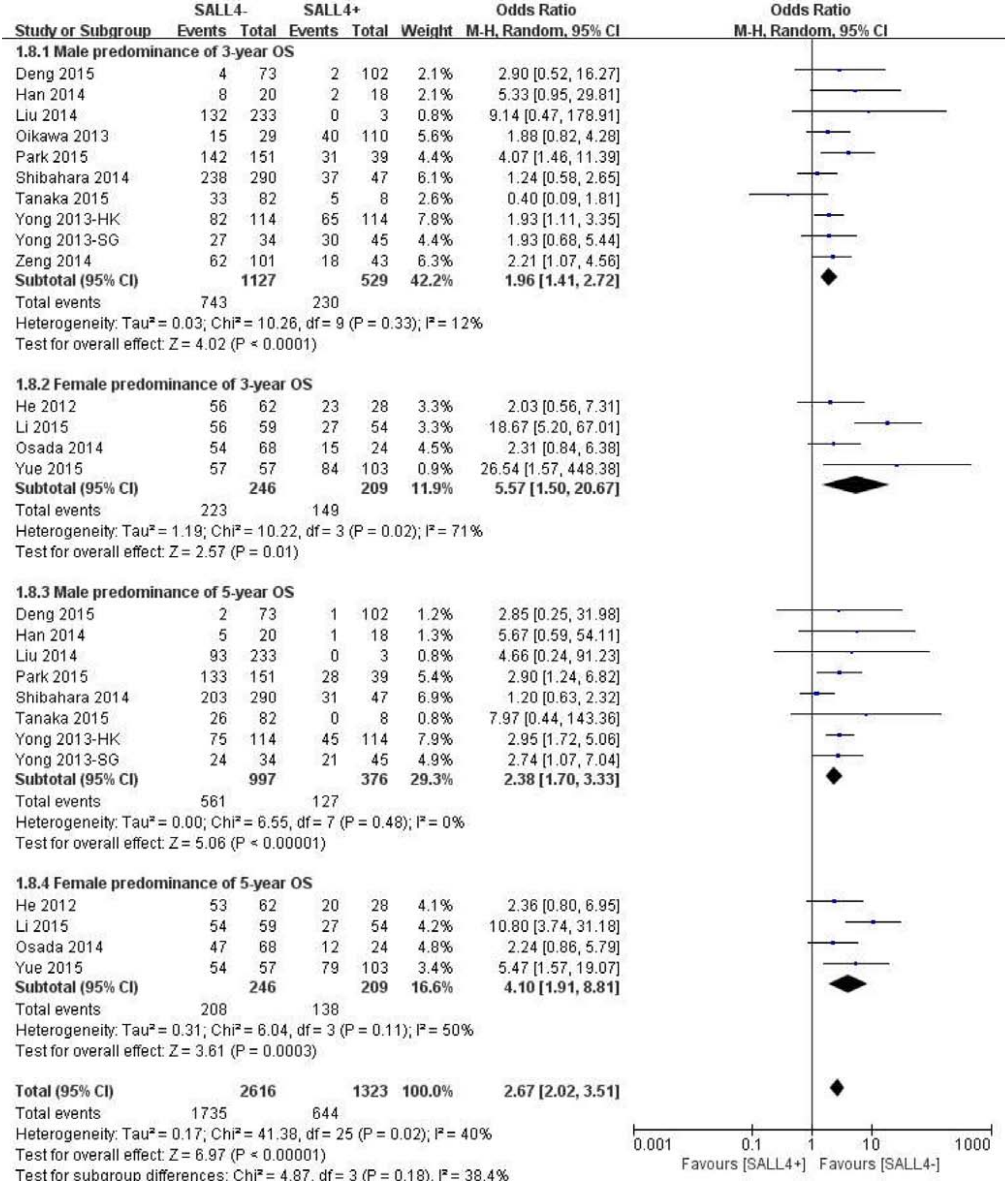

Supplementary Figure S4: The correlation between SALL4 expression and prognosis based on different sex predominance.

Study or Subgroup	ZNF217- Events	ZNF217- Total	ZNF217+ Events	ZNF217+ Total	Weight	Odds Ratio M-H, Random, 95% CI
**2.5.1 Breast cancer of 3-year OS**						
Frietze 2014	145	160	132	159	13.7%	1.98 [1.01, 3.88]
Littlepage 2012	54	59	47	59	5.0%	2.76 [0.90, 8.40]
Nguyen 2014	44	45	52	52	0.6%	0.28 [0.01, 7.11]
Vendrell 2012	30	32	37	46	2.4%	3.65 [0.73, 18.18]
**Subtotal (95% CI)**		**296**		**316**	**21.8%**	**2.17 [1.27, 3.70]**
Total events	273		268			
Heterogeneity: Tau² = 0.00; Chi² = 2.19, df = 3 (P = 0.53); I² = 0%						
Test for overall effect: Z = 2.83 (P = 0.005)						
**2.5.2 Other types of 3-year OS**						
Li 2014	4	18	2	26	1.9%	3.43 [0.56, 21.18]
Li 2015	27	27	50	55	0.7%	5.99 [0.32, 112.41]
Mao 2011	34	62	7	22	5.9%	2.60 [0.93, 7.27]
Rahman 2012	31	40	14	20	4.3%	1.48 [0.44, 4.95]
Rooney 2004	33	43	40	57	7.6%	1.40 [0.57, 3.47]
**Subtotal (95% CI)**		**190**		**180**	**20.4%**	**1.94 [1.12, 3.37]**
Total events	129		113			
Heterogeneity: Tau² = 0.00; Chi² = 1.96, df = 4 (P = 0.74); I² = 0%						
Test for overall effect: Z = 2.35 (P = 0.02)						
**2.5.3 Breast cancer of 5-year OS**						
Frietze 2014	136	160	127	159	18.4%	1.43 [0.80, 2.55]
Littlepage 2012	49	59	35	59	8.5%	3.36 [1.43, 7.91]
Nguyen 2014	43	45	48	52	2.0%	1.79 [0.31, 10.27]
Vendrell 2012	28	32	21	46	4.3%	8.33 [2.52, 27.60]
**Subtotal (95% CI)**		**296**		**316**	**33.3%**	**2.78 [1.25, 6.20]**
Total events	256		231			
Heterogeneity: Tau² = 0.39; Chi² = 7.88, df = 3 (P = 0.05); I² = 62%						
Test for overall effect: Z = 2.50 (P = 0.01)						
**2.5.4 Other types of 5-year OS**						
Li 2014	1	18	0	26	0.6%	4.54 [0.17, 117.99]
Li 2015	20	27	28	55	6.1%	2.76 [1.00, 7.57]
Mao 2011	26	62	5	22	5.0%	2.46 [0.80, 7.51]
Rahman 2012	30	40	11	20	4.8%	2.45 [0.79, 7.64]
Rooney 2004	33	43	34	57	8.0%	2.23 [0.92, 5.40]
**Subtotal (95% CI)**		**190**		**180**	**24.5%**	**2.49 [1.50, 4.12]**
Total events	110		78			
Heterogeneity: Tau² = 0.00; Chi² = 0.23, df = 4 (P = 0.99); I² = 0%						
Test for overall effect: Z = 3.54 (P = 0.0004)						
**Total (95% CI)**		**972**		**992**	**100.0%**	**2.22 [1.73, 2.86]**
Total events	768		690			
Heterogeneity: Tau² = 0.00; Chi² = 12.67, df = 17 (P = 0.76); I² = 0%						
Test for overall effect: Z = 6.28 (P < 0.00001)						
Test for subgroup differences: Chi² = 0.71, df = 3 (P = 0.87), I² = 0%						

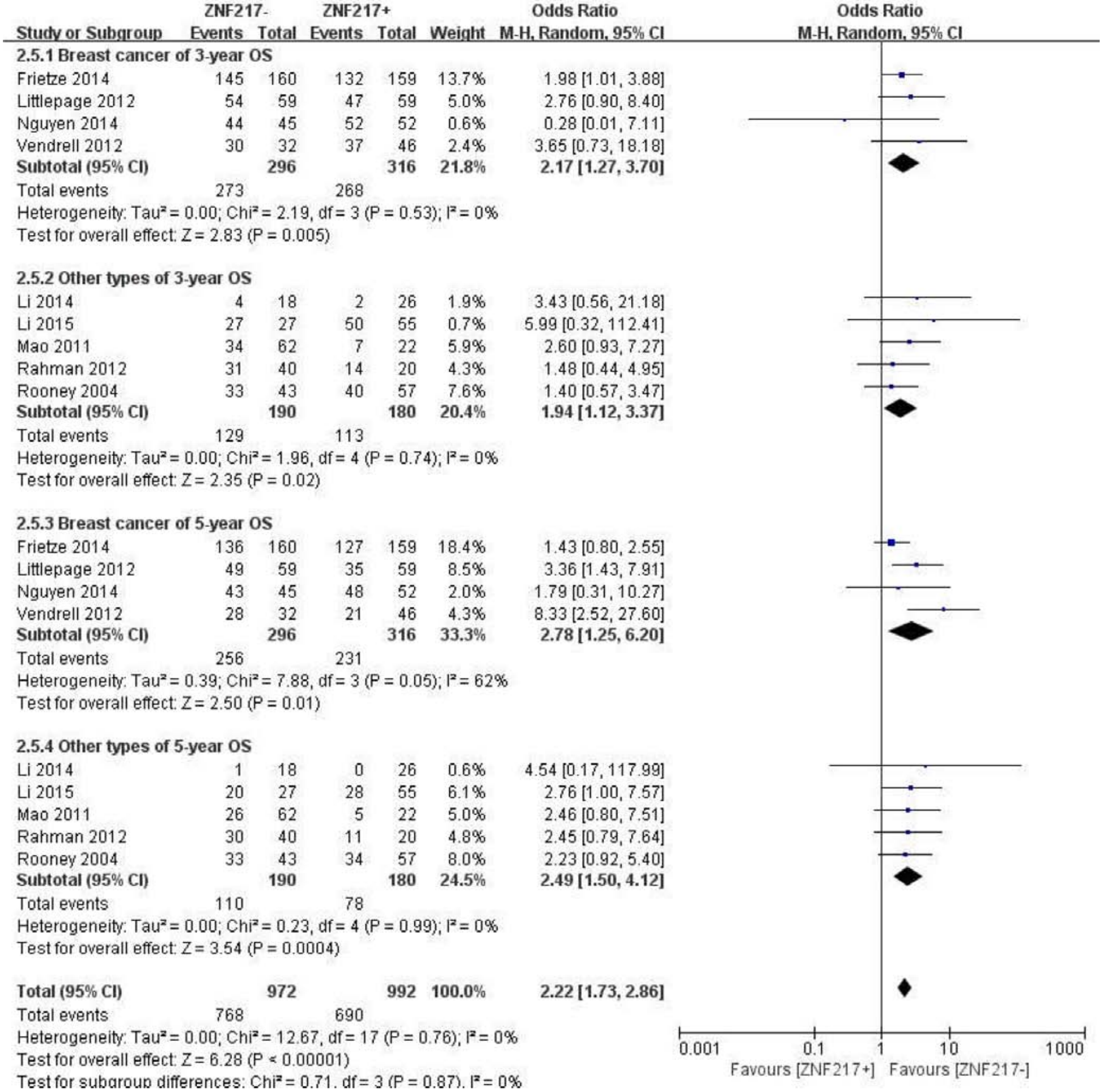

Supplementary Figure S5: The correlation between ZNF217 expression and prognosis based on different cancer types.

Study or Subgroup	ZNF217- Events	ZNF217- Total	ZNF217+ Events	ZNF217+ Total	Weight	Odds Ratio M-H, Fixed, 95% CI
**2.6.1 Mean-age>50 of 3-year OS**						
Li 2015	27	27	50	55	1.3%	5.99 [0.32, 112.41]
Rahman 2012	31	40	14	20	9.1%	1.48 [0.44, 4.95]
Rooney 2004	33	43	40	57	17.3%	1.40 [0.57, 3.47]
Vendrell 2012	30	32	37	46	4.1%	3.65 [0.73, 18.18]
**Subtotal (95% CI)**		**142**		**178**	**31.8%**	**1.90 [1.02, 3.56]**
Total events	121		141			
Heterogeneity: Chi² = 1.82, df = 3 (P = 0.61); I² = 0%						
Test for overall effect: Z = 2.01 (P = 0.04)						
**2.6.2 Mean-age<50 of 3-year OS**						
Li 2014	4	18	2	26	2.8%	3.43 [0.56, 21.18]
Mao 2011	34	62	7	22	10.1%	2.60 [0.93, 7.27]
Nguyen 2014	44	45	52	52	3.4%	0.28 [0.01, 7.11]
**Subtotal (95% CI)**		**125**		**100**	**16.3%**	**2.25 [0.98, 5.17]**
Total events	82		61			
Heterogeneity: Chi² = 1.87, df = 2 (P = 0.39); I² = 0%						
Test for overall effect: Z = 1.91 (P = 0.06)						
**2.6.3 Mean-age>50 of 5-year OS**						
Li 2015	20	27	28	55	10.3%	2.76 [1.00, 7.57]
Rahman 2012	30	40	11	20	7.9%	2.45 [0.79, 7.64]
Rooney 2004	33	43	34	57	14.7%	2.23 [0.92, 5.40]
Vendrell 2012	28	32	21	46	4.7%	8.33 [2.52, 27.60]
**Subtotal (95% CI)**		**142**		**178**	**37.6%**	**3.18 [1.91, 5.28]**
Total events	111		94			
Heterogeneity: Chi² = 3.38, df = 3 (P = 0.34); I² = 11%						
Test for overall effect: Z = 4.46 (P < 0.00001)						
**2.6.4 Mean-age<50 of 5-year OS**						
Li 2014	1	18	0	26	0.8%	4.54 [0.17, 117.99]
Mao 2011	26	62	5	22	9.3%	2.46 [0.80, 7.51]
Nguyen 2014	43	45	48	52	4.3%	1.79 [0.31, 10.27]
**Subtotal (95% CI)**		**125**		**100**	**14.4%**	**2.38 [0.97, 5.86]**
Total events	70		53			
Heterogeneity: Chi² = 0.26, df = 2 (P = 0.88); I² = 0%						
Test for overall effect: Z = 1.88 (P = 0.06)						
**Total (95% CI)**		**534**		**556**	**100.0%**	**2.51 [1.80, 3.49]**
Total events	384		349			
Heterogeneity: Chi² = 8.97, df = 13 (P = 0.77); I² = 0%						
Test for overall effect: Z = 5.44 (P < 0.00001)						
Test for subgroup differences: Chi² = 1.66, df = 3 (P = 0.65), I² = 0%						

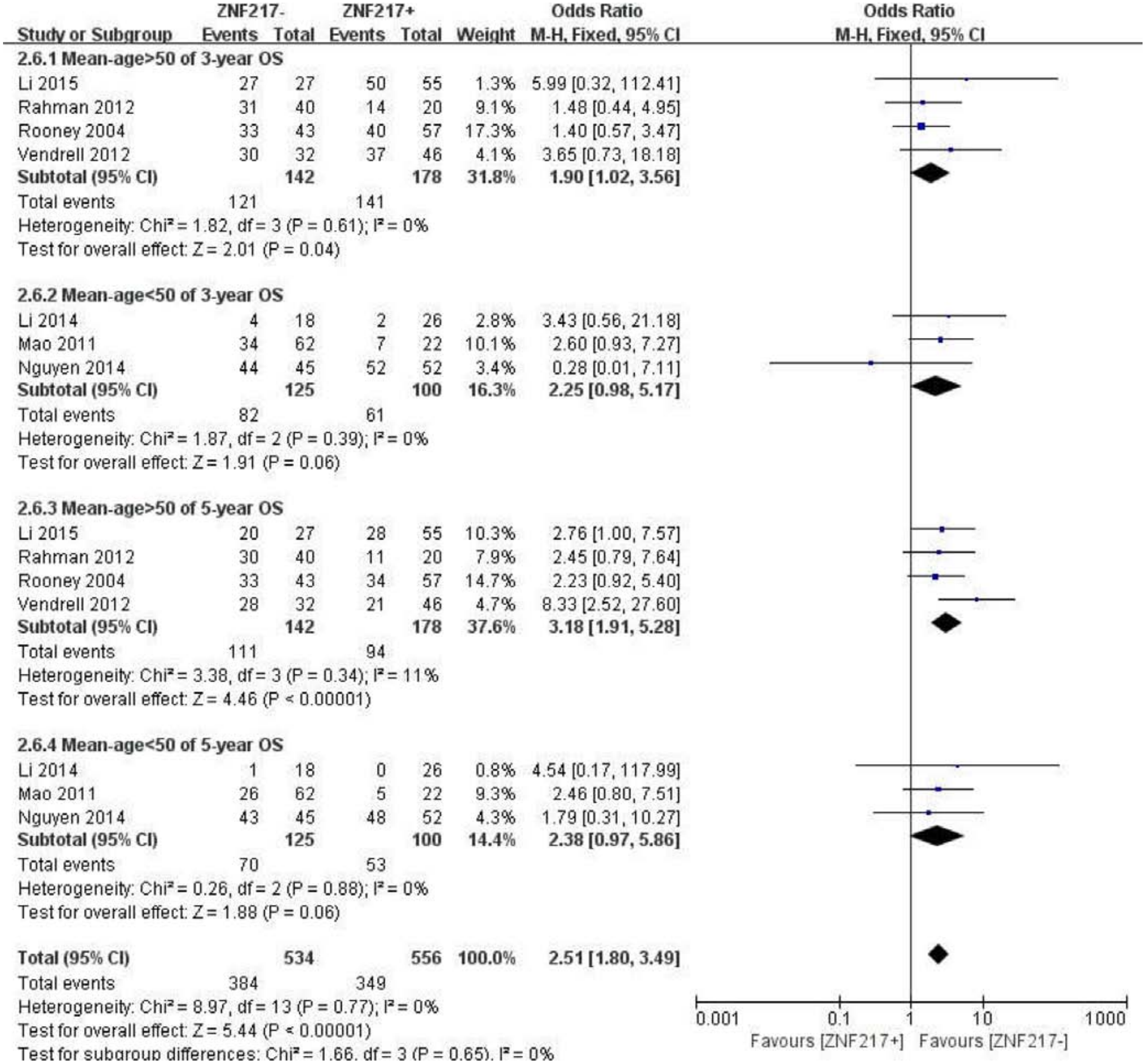

Supplementary Figure S6: The correlation between ZNF217 expression and prognosis based on different mean-age.

Study or Subgroup	ZNF217- Events	ZNF217- Total	ZNF217+ Events	ZNF217+ Total	Weight	Odds Ratio M-H, Random, 95% CI
**2.7.1 Asian studies of 3-year OS**						
Li 2014	4	18	2	26	1.9%	3.43 [0.56, 21.18]
Li 2015	27	27	50	55	0.7%	5.99 [0.32, 112.41]
Mao 2011	34	62	7	22	5.9%	2.60 [0.93, 7.27]
Rahman 2012	31	40	14	20	4.3%	1.48 [0.44, 4.95]
**Subtotal (95% CI)**		**147**		**123**	**12.8%**	**2.35 [1.17, 4.73]**
Total events	96		73			
Heterogeneity: Tau² = 0.00; Chi² = 1.17, df = 3 (P = 0.76); I² = 0%						
Test for overall effect: Z = 2.40 (P = 0.02)						
**2.7.2 Non-Asian studies of 3-year OS**						
Frietze 2014	145	160	132	159	13.7%	1.98 [1.01, 3.88]
Littlepage 2012	54	59	47	59	5.0%	2.76 [0.90, 8.40]
Nguyen 2014	44	45	52	52	0.6%	0.28 [0.01, 7.11]
Rooney 2004	33	43	40	57	7.6%	1.40 [0.57, 3.47]
Vendrell 2012	30	32	37	46	2.4%	3.65 [0.73, 18.18]
**Subtotal (95% CI)**		**339**		**373**	**29.4%**	**1.94 [1.22, 3.07]**
Total events	306		308			
Heterogeneity: Tau² = 0.00; Chi² = 2.84, df = 4 (P = 0.58); I² = 0%						
Test for overall effect: Z = 2.81 (P = 0.005)						
**2.7.3 Asian studies of 5-year OS**						
Li 2014	1	18	0	26	0.6%	4.54 [0.17, 117.99]
Li 2015	20	27	28	55	6.1%	2.76 [1.00, 7.57]
Mao 2011	26	62	5	22	5.0%	2.46 [0.80, 7.51]
Rahman 2012	30	40	11	20	4.8%	2.45 [0.79, 7.64]
**Subtotal (95% CI)**		**147**		**123**	**16.5%**	**2.62 [1.42, 4.84]**
Total events	77		44			
Heterogeneity: Tau² = 0.00; Chi² = 0.14, df = 3 (P = 0.99); I² = 0%						
Test for overall effect: Z = 3.07 (P = 0.002)						
**2.7.4 Non-Asian studies of 5-year OS**						
Frietze 2014	136	160	127	159	18.4%	1.43 [0.80, 2.55]
Littlepage 2012	49	59	35	59	8.5%	3.36 [1.43, 7.91]
Nguyen 2014	43	45	48	52	2.0%	1.79 [0.31, 10.27]
Rooney 2004	33	43	34	57	8.0%	2.23 [0.92, 5.40]
Vendrell 2012	28	32	21	46	4.3%	8.33 [2.52, 27.60]
**Subtotal (95% CI)**		**339**		**373**	**41.3%**	**2.58 [1.42, 4.67]**
Total events	289		265			
Heterogeneity: Tau² = 0.21; Chi² = 7.87, df = 4 (P = 0.10); I² = 49%						
Test for overall effect: Z = 3.12 (P = 0.002)						
**Total (95% CI)**		**972**		**992**	**100.0%**	**2.22 [1.73, 2.86]**
Total events	768		690			
Heterogeneity: Tau² = 0.00; Chi² = 12.67, df = 17 (P = 0.76); I² = 0%						
Test for overall effect: Z = 6.28 (P < 0.00001)						
Test for subgroup differences: Chi² = 0.85, df = 3 (P = 0.84), I² = 0%						

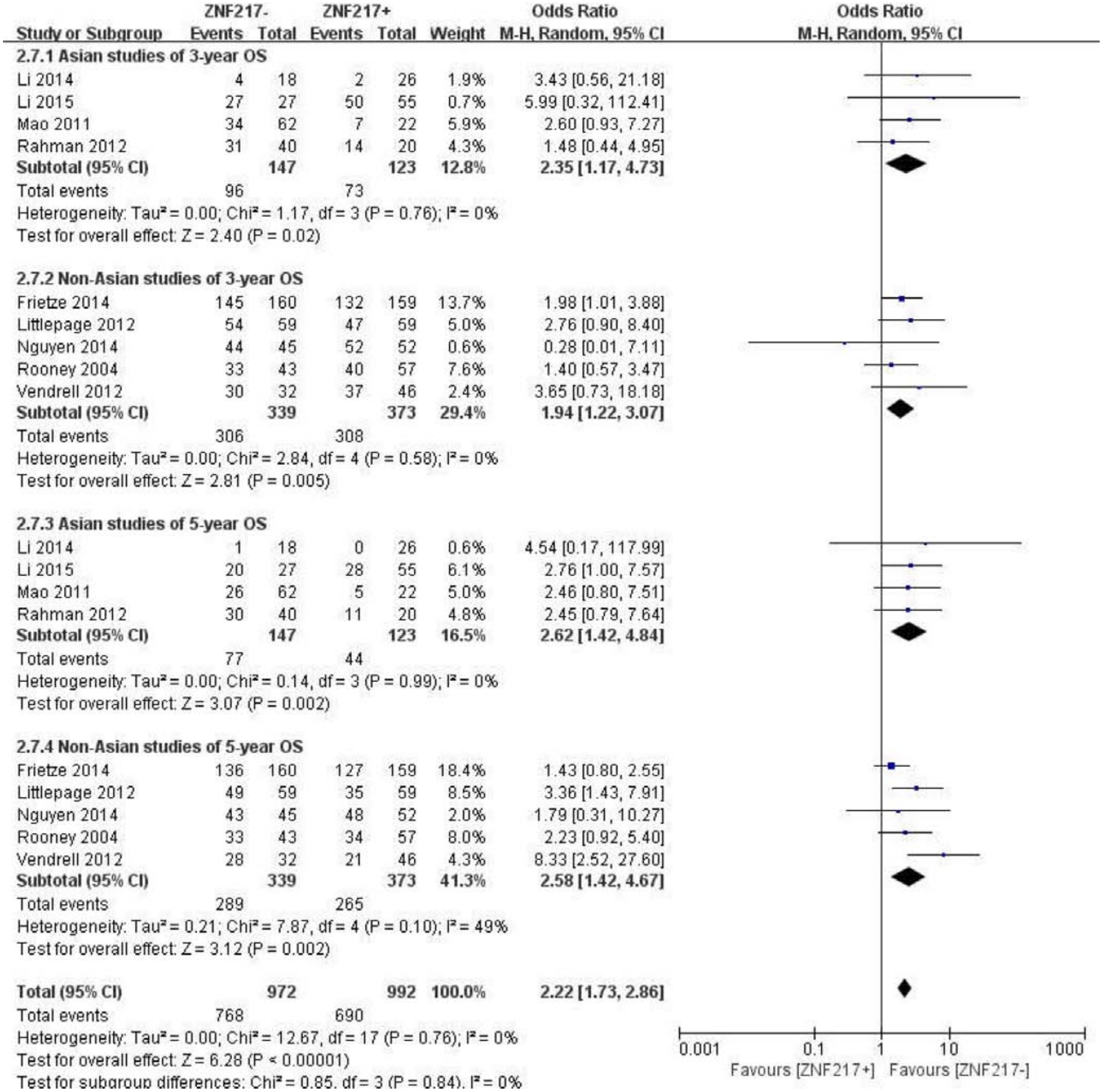

Supplementary Figure S7: The correlation between ZNF217 expression and prognosis based on different source regions.

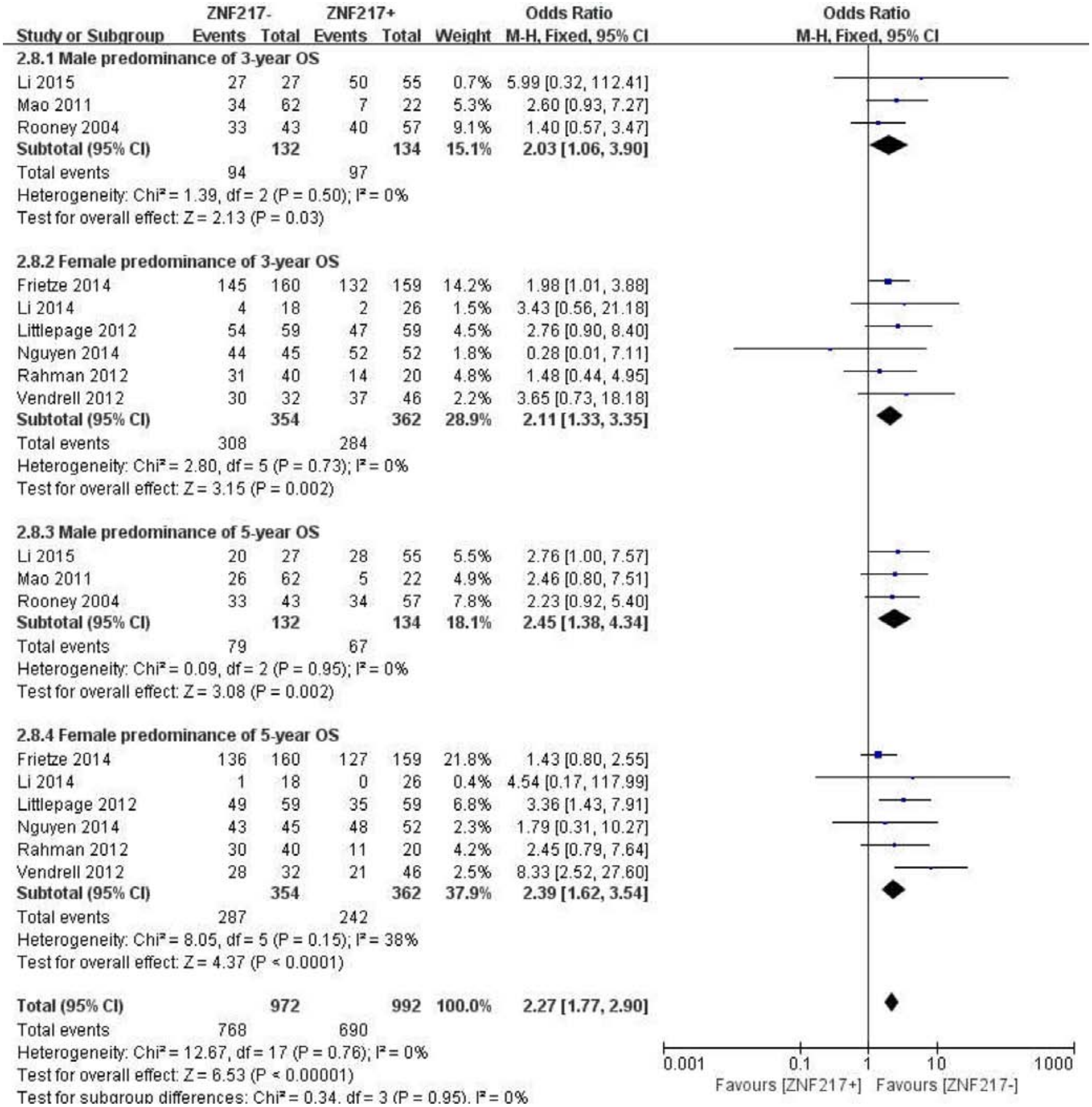

Study or Subgroup	ZNF217- Events	ZNF217- Total	ZNF217+ Events	ZNF217+ Total	Weight	Odds Ratio M-H, Fixed, 95% CI
**2.8.1 Male predominance of 3-year OS**						
Li 2015	27	27	50	55	0.7%	5.99 [0.32, 112.41]
Mao 2011	34	62	7	22	5.3%	2.60 [0.93, 7.27]
Rooney 2004	33	43	40	57	9.1%	1.40 [0.57, 3.47]
**Subtotal (95% CI)**		**132**		**134**	**15.1%**	**2.03 [1.06, 3.90]**
Total events	94		97			
Heterogeneity: Chi² = 1.39, df = 2 (P = 0.50); I² = 0%						
Test for overall effect: Z = 2.13 (P = 0.03)						
**2.8.2 Female predominance of 3-year OS**						
Frietze 2014	145	160	132	159	14.2%	1.98 [1.01, 3.88]
Li 2014	4	18	2	26	1.5%	3.43 [0.56, 21.18]
Littlepage 2012	54	59	47	59	4.5%	2.76 [0.90, 8.40]
Nguyen 2014	44	45	52	52	1.8%	0.28 [0.01, 7.11]
Rahman 2012	31	40	14	20	4.8%	1.48 [0.44, 4.95]
Vendrell 2012	30	32	37	46	2.2%	3.65 [0.73, 18.18]
**Subtotal (95% CI)**		**354**		**362**	**28.9%**	**2.11 [1.33, 3.35]**
Total events	308		284			
Heterogeneity: Chi² = 2.80, df = 5 (P = 0.73); I² = 0%						
Test for overall effect: Z = 3.15 (P = 0.002)						
**2.8.3 Male predominance of 5-year OS**						
Li 2015	20	27	28	55	5.5%	2.76 [1.00, 7.57]
Mao 2011	26	62	5	22	4.9%	2.46 [0.80, 7.51]
Rooney 2004	33	43	34	57	7.8%	2.23 [0.92, 5.40]
**Subtotal (95% CI)**		**132**		**134**	**18.1%**	**2.45 [1.38, 4.34]**
Total events	79		67			
Heterogeneity: Chi² = 0.09, df = 2 (P = 0.95); I² = 0%						
Test for overall effect: Z = 3.08 (P = 0.002)						
**2.8.4 Female predominance of 5-year OS**						
Frietze 2014	136	160	127	159	21.8%	1.43 [0.80, 2.55]
Li 2014	1	18	0	26	0.4%	4.54 [0.17, 117.99]
Littlepage 2012	49	59	35	59	6.8%	3.36 [1.43, 7.91]
Nguyen 2014	43	45	48	52	2.3%	1.79 [0.31, 10.27]
Rahman 2012	30	40	11	20	4.2%	2.45 [0.79, 7.64]
Vendrell 2012	28	32	21	46	2.5%	8.33 [2.52, 27.60]
**Subtotal (95% CI)**		**354**		**362**	**37.9%**	**2.39 [1.62, 3.54]**
Total events	287		242			
Heterogeneity: Chi² = 8.05, df = 5 (P = 0.15); I² = 38%						
Test for overall effect: Z = 4.37 (P < 0.0001)						
**Total (95% CI)**		**972**		**992**	**100.0%**	**2.27 [1.77, 2.90]**
Total events	768		690			
Heterogeneity: Chi² = 12.67, df = 17 (P = 0.76); I² = 0%						
Test for overall effect: Z = 6.53 (P < 0.00001)						
Test for subgroup differences: Chi² = 0.34, df = 3 (P = 0.95), I² = 0%						

Supplementary Figure S8: The correlation between ZNF217 expression and prognosis based on different sex predominance.
